# Assessment of *in vivo* microstructure alterations in gray matter using DKI in internet gaming addiction

**DOI:** 10.1186/1744-9081-10-37

**Published:** 2014-10-24

**Authors:** Yawen Sun, Jinhua Sun, Yan Zhou, Weina Ding, Xue Chen, Zhiguo Zhuang, Jianrong Xu, Yasong Du

**Affiliations:** Department of Radiology, Ren Ji Hospital, School of Medicine, Shanghai Jiao Tong University, Shanghai, 200127 P.R. China; Department of Child and Adolescent Psychiatry, Shanghai Mental Health Center, Shanghai Jiao Tong University, Shanghai, 200030 P.R. China

**Keywords:** Internet gaming addiction, Diffusional kurtosis imaging, Gray matter, Posterior cingulate cortex

## Abstract

**Background:**

The aim of the current study was to investigate the utility of diffusional kurtosis imaging (DKI) in the detection of gray matter (GM) alterations in people suffering from Internet Gaming Addiction (IGA).

**Methods:**

DKI was applied to 18 subjects with IGA and to 21 healthy controls (HC). Whole-brain voxel-based analyses were performed with the following derived parameters: mean kurtosis metrics (MK), radial kurtosis (K_⊥_), and axial kurtosis (K_//_). A significance threshold was set at *P* <0.05, AlphaSim corrected. Pearson’s correlation was performed to investigate the correlations between the Chen Internet Addiction Scale (CIAS) and the DKI-derived metrics of regions that differed between groups. Additionally, we used voxel-based morphometry (VBM) to detect GM-volume differences between the two groups.

**Results:**

Compared with the HC group, the IGA group demonstrated diffusional kurtosis parameters that were significantly less in GM of the right anterolateral cerebellum, right inferior and superior temporal gyri, right supplementary motor area, middle occipital gyrus, right precuneus, postcentral gyrus, right inferior frontal gyrus, left lateral lingual gyrus, left paracentral lobule, left anterior cingulate cortex, and median cingulate cortex. The bilateral fusiform gyrus, insula, posterior cingulate cortex (PCC), and thalamus also exhibited less diffusional kurtosis in the IGA group. MK in the left PCC and K_⊥_ in the right PCC were positively correlated with CIAS scores. VBM showed that IGA subjects had higher GM volume in the right inferior and middle temporal gyri, and right parahippocampal gyrus, and lower GM volume in the left precentral gyrus.

**Conclusions:**

The lower diffusional kurtosis parameters in IGA suggest multiple differences in brain microstructure, which may contribute to the underlying pathophysiology of IGA. DKI may provide sensitive imaging biomarkers for assessing IGA severity.

## Introduction

Internet use is regarded as an essential element of modern life and the number of internet users is constantly increasing. At the same time, percentages of excessive users are also on the rise [1]. One recent study, based on 24,013 fourth- to ninth-grade students recruited from 100 counties in 31 provinces in China, reported that 11.7% of the students were internet users, with a prevalence of Internet Addiction (IA) of 6.3% [2]. IA consists of at least three subtypes: Internet Gaming Addiction (IGA), Sexual Preoccupations, and Email/Text Messaging Addiction [3], which may lead to negative consequences in daily life, such as low self-esteem, sense of loneliness, low self-disclosure, anti-social behaviors, stronger suicidal intention, and sensation-seeking [4].

Online games are a mainstream recreation among internet users, and IGA, which broadly refers to the inability to stop playing, has become a severe social issue [1, 5]. The etiology of IGA has yet to be studied in detail [5]. Research indicates that it is associated with a number of risk factors including certain personality traits (neuroticism, aggression and hostility, sensation-seeking) [6, 7], gaming motivations (coping with daily stressors and escapism, online relationships, mastery, control, recognition, completion, excitement, and challenge) [8, 9], and structural characteristics of the game (online vs. offline, positive reinforcement, the enjoyment of particular game features) [10, 11]. Both structural and functional brain abnormalities associated with IGA have been reported in several studies, demonstrating that IGA could result in neuroadaptation and structural alterations as a consequence of prolonged activity in brain areas associated with addiction [12–15].

Diffusional kurtosis imaging (DKI) is an imaging technique that characterizes non-Gaussian water diffusion process [16] and quantifies the apparent diffusion coefficient and apparent diffusional kurtosis. It has been demonstrated that DKI can provide information that diffusion tensor imaging (DTI) does not, particularly regarding microstructures in the brain [17, 18], and thus can potentially improve the sensitivity and specificity of brain-tissue characterization *in vivo*. In addition, DKI permits the characterization of microstructural integrity of both gray and white matter because it is not limited to anisotropic environments [19]. The sensitivity to gray matter (GM) diffusion may be important for the examination of microstructural integrity in the brain. The aim of the present study was to investigate the application of DKI for detecting differences between the GM of an IGA population and normal controls.

## Method and materials

### Theory

Conventional DTI assumes Gaussian (i.e., unrestricted and free) diffusion. The apparent diffusivity (D_app_) is derived by linearly fitting the diffusion-weighted (DW) signals acquired with one or more non-zero b-values to the following linear equation:
1

In DKI [20, 21], both apparent a diffusion coefficient (*D*_*app*_) and an apparent diffusion kurtosis (*K*_*app*_) along each applied diffusion gradient direction are estimated together by fitting the following equation to the multiple DW signals acquired using a range of b-values:
2

where S(b) is the DW signal intensity at a particular b-value, and S(0) the signal without applying any diffusion gradient. MK is defined as the kurtosis averaged across all directions. Note that 15 independent elements are required to construct the 4^th^ order diffusion kurtosis tensor (KT). KT can be transformed to a coordinate system formed by the three orthogonal eigenvectors of the 2^nd^ order DT [22, 23]:
3

The kurtosis along an individual DT eigenvector can be computed from the transformed KT(*Ŵ*):
4

where λ_i_ are the eigenvalues of the DT (λ1 > λ2 > λ3). Axial kurtosis (K_//_) and radial kurtosis (K_⊥_) can then be derived [22, 23]:
5

is the kurtosis along the principal DT eigenvector, and
6

is the average kurtosis along the other two eigenvectors.

### Subject selection

Demographic information such as gender, age, education level, and weekly Internet use were acquired through questionnaires. The present study was approved by the Ethics Committee of Ren Ji Hospital, School of Medicine, Shanghai Jiao Tong University. Full written informed consent was obtained from each participant before MRI examinations.

All subjects were interviewed by a psychiatrist regarding their medical history and underwent a basic physical examination that included blood pressure and heart rate measurements. Following the physical, subjects were assessed for psychiatric disorders using the Mini International Neuropsychiatric Interview (MINI) [24]. Exclusion criteria included a history of substance abuse or dependence, previous hospitalization for psychiatric disorders, or a history of major psychiatric disorders, such as schizophrenia, depression, anxiety disorder, or psychotic episodes.

The diagnostic questionnaire for IGA was adapted from the DSM-IV criteria for IA according to the modified Diagnostic Questionnaire for Internet Addiction criteria (the YDQ) by Beard [25]. IA falls into three subtypes, and only subjects characterized as the IGA subtype were enrolled in this study. Thus, all subjects mostly focused on online gaming when using the Internet. Eighteen subjects who satisfied these criteria were recruited from the Outpatient department of Shanghai Mental Health Center. Twenty-one age- and gender-matched healthy individuals were recruited as the control group through advertisements. All subjects were right-handed non-smokers, and the IGA subjects were currently not receiving any medication or therapy for their addiction.

### Clinical assessments

The Chen Internet Addiction Scale (CIAS) [26], the Self-Rating Anxiety Scale (SAS) [27], the Self-rating Depression Scale (SDS) [28], and the Barratt Impulsiveness Scale-11 (BIS-11) [29] were administered to assess the participants’ clinical features. All questionnaires were translated into Chinese form English for the benefit of the subjects. The cut-off scores for the CIAS, SAS, and SDS were 64, 50, and 53 respectively. The BIS-11 is a 30-item self-report questionnaire that is widely used to measure impulsivity in a variety of populations. Higher scores indicate higher impulsivity.

### MRI acquisition

All scans were acquired on a GE Signa HDxT 3.0 T MRI scanner (General Electric Medical System, Milwaukee, WI, USA) with a standard 8-channel head coil with foam padding. DWI images were acquired with three b-values (0, 1000, and 2000 s/mm^2^) and diffusion encoding vectors along 25 nonparallel directions for each nonzero b-value. A spin-echo echo-planar imaging sequence was applied to acquire DWI images, with the following parameters: repetition time (TR) = 10500 ms, echo time (TE) = 99.3 ms, number of averages = 1, slice thickness = 4 mm, field of view (FOV) = 256 mm × 256 mm, matrix = 128 × 128, gradient length = 30.9 ms, diffusion gradient = 39.1 ms, and scan time = 557 s. Signal to Noise Ratio (SNR) varied between 46 and 26 over the entire b-value range (0–2000 s/mm^2^). Additionally, 3D-Fast Spoiled Gradient Recalled sequence (3D-FSPGR) images were acquired for anatomical reference with the following parameters: TR = 6.1 ms, TE = 2.8 ms, slice thickness = 1 mm, gap = 0, flip angle = 15°, FOV =256 mm × 256 mm, matrix = 256 × 256, number of slices = 166, and scan time = 334 s. Axial T1 and T2W-weighted sequences were also acquired with the following parameters: T1-weighted fast-field echo sequences (TR = 331 ms, TE = 4.6 ms, FOV = 256 mm × 256 mm, 34 slices, voxel size = 0.5 mm × 0.5 mm × 4 mm, and scan time = 275 s); T2W turbo spin-echo sequences (TR = 3013 ms, TE = 80 ms, FOV = 256 mm × 256 mm, 34 slices, voxel size = 0.5 mm × 0.5 mm × 4 mm, and scan time = 217 s).

### Data analysis

After confirming that the variance within each group was homogenous, two-sample t-tests were performed to determine the demographic differences between groups, and a chai-squared test was applied for gender comparison. Two-tailed p-values ≤0.05 were considered statistically significant for all analyses. All statistical analyses were performed using SPSS software (v.17.0.1, IBM, USA). T1- and T2-weighted images were inspected by two experienced neuroradiologists together. No gross abnormalities were observed in either group.

For all DWI images, raw DWI data-distortion induced by eddy currents were corrected using the “eddy correct” tool in FSL (FMRIB Software Library, Oxford, UK), and non-brain tissue was removed from the image using the BET tool in FSL. Axial kurtosis (K_//_) was derived from the diffusion tensor, while radial kurtosis (K_⊥_), and mean kurtosis (MK) were derived from the kurtosis tensor. We used the DKI processing toolbox available with Functool software version 9.4.05a (GE workstation Advantage Windows 4.4) to calculate K_//_ from the apparent diffusion, and MK and K_⊥_ from the kurtosis coefficients. Voxel-based analysis (VBA) was performed using Statistical Parametric Mapping (SPM8, Wellcome Department of Imaging Neuroscience, London, UK; available at http://www.fil.ion.ucl.ac.uk/spm/software/spm8) implemented on MATLAB R2010a (MathWorks Inc., Sherborn, MA, USA). Implementation was as follows: primarily, a participant-specific b0 template was generated based on all participant data. Each b0 volume was normalized to the EPI template provided by SPM8 using a nonlinear co-registration method, with a reslicing resolution of 2 mm × 2 mm × 2 mm. The normalized b0 templates were averaged and smoothed with a 6 mm full-width-at-half-maximum (FWHM) Gaussian kernel to yield the b0 template. Second, all original b0 images from each subject were normalized to the b0 template with a reslicing resolution of 2 mm × 2 mm × 2 mm, and the resulting transformation matrix was applied to the MK, K_//_, and K_⊥_ maps. All acquired images were smoothed with a 6 mm FWHM isotropic Gaussian kernel to decrease spatial noise and to compensate for the inexact nature of normalization. In addition, a GM mask was generated from the T1-weighted (3D- FSPGR) image, which was segmented following the SPM8 segmentation routine. For each subject, the T1-weighted (3D- FSPGR) image was segmented into GM, white matter (WM) and cerebrospinal fluid, which were then normalized to the standard Montreal Neurological Institute (MNI) space using SPM8 with a reslicing resolution of 2 mm × 2 mm × 2 mm. The normalized GM images for each subject were averaged and smoothed with a 6 mm FWHM Gaussian kernel, the mean of which, including all voxels with a GM probability greater than 0.5, was converted into binary masks for further analyses.

### Statistical analysis

VBA was performed for the entire brain on normalized and smoothed MK, K_//_, and K_⊥_ maps. Two-sample t-tests were applied to detect GM differences between groups. Correction for multiple comparisons was performed using AlphaSim software, as determined by Monte Carlo simulations. Statistical maps derived from the two-sample t-tests were created using a combined threshold of *P* <0.001 and a minimum cluster size of 13 voxels, yielding a corrected threshold of *P* <0.05.

### Correlations between GM differences in microstructure and severity of IGA

The brain regions shown to have significantly different MK, K_⊥_ or K_//_ values in the IGA group compared with those of the control group were extracted as the region of interest (ROI) masks. These ROI masks were then projected onto the normalized and smoothed images of the 18 IGA subjects, and the MK, K_⊥_, and K_//_ values were calculated for the ROIs. Next, analysis was performed to investigate the correlation between these values and CIAS scores, which measure the severity of IGA. Correlations were judged significant at *P* <0.05.

### Voxel-based morphometry (VBM) analysis

Three-dimensional geometric correction was performed during reconstruction of the images. Image segmentation and registration were performed using the segmentation algorithm and the DARTEL registration algorithm incorporated in SPM8. Following the method described by van Holst [30], we used the Display function of SPM8 to manually set the image-space origin to the anterior commissure and align each image with the plane of the anterior and posterior commissures. Then, the individual native images of all participants were segmented into GM, white matter and cerebrospinal fluid (CSF). Next, GM and white matter segments were registered to a template generated from their own mean by DARTEL, before normalizing them to the Montreal Neurological Institute template space. Then, DARTEL registrations were performed with default parameter settings. Finally, we modulated the final outputs (i.e., preserving the total amount of gray matter from the original image), and smoothed the GM images with an 8-mm full-width at half-maximum Gaussian kernel. Voxel-wise comparisons of GM volume were performed between the groups using a two-sample t-test with SPM8. The significance of group differences was estimated by the theory of random Gaussian fields, and significance levels were set at uncorrected *P* <0.001 and the cluster size was set at >100 voxels.

## Results

### Demographic and clinical measurements

The demographic and behavioral measurements for the IGA and control subjects are shown in Tables 1 and 2. No significant between-group differences in the distributions of age, gender, or education level were observed. Compared with the controls, the IGA group possessed higher CIAS, SAS, SDS, and BIS-11 scores (*P* = 0.000).

**Table 1:** **Demographic and personality characteristics of the study participants**

	IGA group (n = 18)	Control group (n = 21)	p-value
	(Mean ± SD)	(Mean ± SD)	
Age (years)	20.5 ± 3.55	21.95 ± 2.39	0.138
Gender (M/F)	15/3	18/3	0.06
Education (years)	11.39 ± 1.85	12.38 ± 2.13	0.13
Chen Internet Addiction Scale (CIAS)	74.44 ± 8.33	38.43 ± 9.10	<0.0001
Self-Rating Anxiety Scale (SAS)	53.66 ± 9.71	40.95 ± 8.44	<0.0001
Self-rating depression scale (SDS)	54.72 ± 10.44	38.57 ± 6.67	<0.0001
Barratt Impulsiveness Scale-11 (BIS-11)	63.94 ± 8.26	50.81 ± 6.95	<0.0001

Two-sample t-test was used for group comparisons. Chi-square test was used for gender comparison.

*Abbreviations*: *IGA* internet gaming addiction, *SD* standard deviation.

**Table 2:** **Demographic data, CIAS, SAS, SDS, and BIS-11 scores for all participants**

	Sex	Age	Eyr	CIAS	SAS	SDS	BIS-11			
							Attentional key	Motor key	Non-planning key	Total score
IGA1	M	20	14	64	64	61	19	24	28	71
IGA2	M	14	12	65	56	54	18	22	26	66
IGA3	M	19	12	64	61	59	12	17	23	52
IGA4	M	15	8	66	54	61	12	17	22	51
IGA5	F	22	12	76	58	48	14	17	25	56
IGA6	M	17	9	83	50	54	19	24	29	72
IGA7	M	22	16	84	51	49	13	16	27	56
IGA8	M	18	10	75	46	61	15	18	31	64
IGA9	M	23	11	87	56	68	23	24	29	76
IGA10	M	23	12	81	59	36	23	23	27	73
IGA11	M	22	12	65	30	56	13	22	26	61
IGA12	M	24	13	64	44	34	15	20	26	61
IGA13	M	16	10	75	70	70	15	33	27	65
IGA14	M	22	12	86	68	40	20	27	33	80
IGA15	M	27	11	76	43	50	16	22	26	64
IGA16	F	24	10	78	48	56	16	24	24	64
IGA17	F	23	11	82	58	60	16	22	27	65
IGA18	M	18	10	69	50	61	15	14	25	54
CON1	F	25	11	32	31	28	11	13	19	43
CON2	M	18	9	39	41	46	13	20	21	54
CON3	F	26	9	30	39	33	10	17	19	46
CON4	M	27	9	26	40	38	12	13	19	44
CON5	M	23	11	50	55	44	18	16	26	60
CON6	M	21	13	26	29	28	10	11	16	37
CON7	M	21	13	52	36	35	13	18	25	56
CON8	M	22	14	32	34	39	10	15	19	44
CON9	M	22	14	51	46	46	19	18	26	63
CON10	M	21	14	34	35	33	10	18	18	46
CON11	M	18	9	31	46	38	11	18	20	49
CON12	F	23	16	50	48	45	18	18	26	62
CON13	M	22	15	47	41	39	12	15	25	52
CON14	M	24	15	44	30	28	12	17	19	48
CON15	M	23	14	40	35	39	10	19	22	51
CON16	M	22	14	32	28	33	15	16	22	53
CON17	M	22	12	42	46	44	14	16	22	52
CON18	M	19	12	28	43	34	10	17	15	42
CON19	M	19	12	28	53	44	11	18	30	59
CON20	M	20	12	47	48	46	12	14	25	51
CON21	M	23	12	46	56	50	15	20	20	55

*Abbreviations*: *Eyr* Educational years, *SAS* Self-Rating Anxiety Scale, *CIAS* Chen Internet Addiction Scale, *SDS* Self-rating depression scale, *BIS-11* Barratt Impulsiveness Scale-11.

### Between-group analysis of DKI parameters

**Figure 1:**
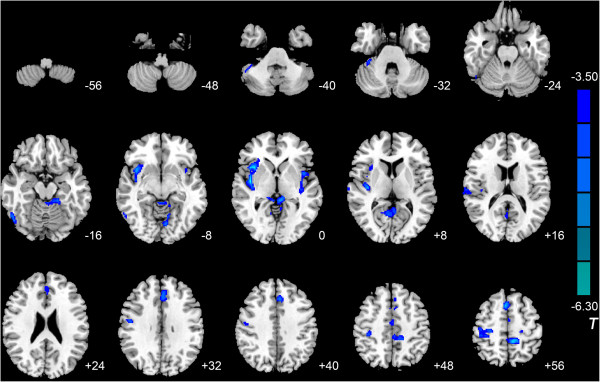
**Significant differences in MK between IGA and HC subjects.** Compared with the control group, IGA subjects exhibited lower MK in the right anterior lobe of the cerebellum, left fusiform gyrus, left lingual gyrus, right inferior temporal gyrus, bilateral insula, left posterior cingulate cortex, right superior temporal gyrus, right precentral gyrus, left paracentral lobule, left anterior cingulate gyrus, left median cingulate gyrus, and right supplementary motor area (*P* <0.05, AlphaSim-corrected). T-values are color-coded on the right. Blue indicates IGA group < HC. *The left section of the figure represents the patient’s right side. *HC, healthy control. IGA, internet gaming addiction. MK, kurtosis metrics.

**Figure 2:**
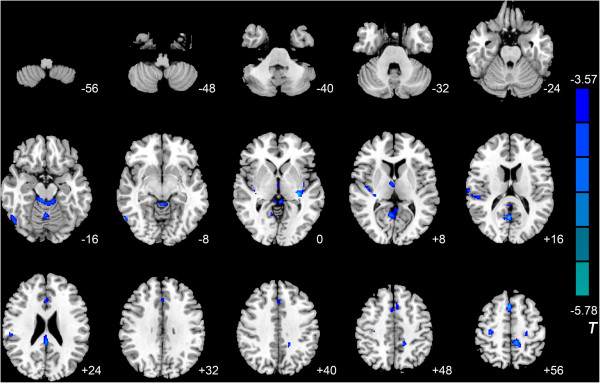
**Significant differences in axial kurtosis (K**_**//**_ **) between IGA and HC subjects.** Compared with the control group, the IGA group exhibited lower K_//_ in the right middle occipital gyrus, right anterior lobe of the cerebellum, right precuneus, right insula, bilateral thalamus, right postcentral gyrus, right posterior cingulate cortex, right supplementary motor area, left median cingulate gyrus, and bilateral precentral gyrus (*P* <0.05, AlphaSim-corrected). T-values are color-coded on the right. Blue indicates IGA group < HC. *The left section of the figure represents the patient’s right side. *HC, healthy control. IGA, internet gaming addiction.

**Figure 3:**
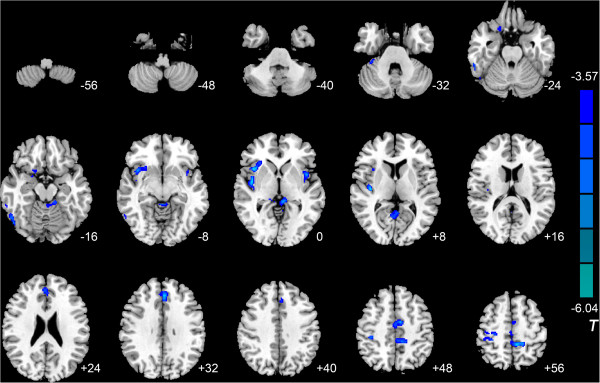
**Significant differences in radial kurtosis (K**_**⊥**_ **) between IGA and HC subjects.** Compared with the control group, the IGA group exhibited lower K_⊥_ in the right inferior temporal gyrus, right orbitofrontal cortex, right fusiform gyrus, bilateral insula, left anterior cingulate cortex, left median cingulate gyrus, right postcentral gyrus, left paracentral lobule, and right posterior cingulate cortex (*P* <0.05, AlphaSim-corrected). T-values are color-coded on the right. Blue indicates IGA group < HC. *The left part of the figure represents the patient’s right side. *HC, healthy control. IGA, internet gaming addiction.

Compared with the control group, the IGA group exhibited lower MK in the right anterior lobe of the cerebellum, left fusiform gyrus, left lingual gyrus, right inferior temporal gyrus, bilateral insula, left posterior cingulate cortex (PCC), right superior temporal gyrus, right precentral gyrus, left paracentral lobule, left anterior cingulate cortex (ACC), left median cingulate gyrus, and right supplementary motor area (SMA). Lower K_//_ was observed in the right middle occipital gyrus, right anterior lobe of the cerebellum, right precuneus, right insula, bilateral thalamus, right postcentral gyrus, right PCC, right SMA, left middle cingulate cortex (MCC), and bilateral precentral gyrus, and lower K_⊥_ was observed in the right inferior temporal gyrus, right orbitofrontal cortex (OFC), right fusiform gyrus, bilateral insula, left ACC, left MCC, right postcentral gyrus, left paracentral lobule, and right PCC (Tables 3, 4 and 5, Figures 1, 2, and 3) No areas showed higher MK, K_//_, or K_⊥_.

**Table 3:** **Summary of MK changes between the IGA and control groups**

	Peak MNI coordinate region	Peak MNI coordinates			Number of cluster voxels	Peak ***t*** value
		x	y	z		
1	Right cerebellum anterior lobe	42	−42	−28	150	−5.33
2	Left fusiform gyrus	−6	−36	0	414	−5.75
3	Left lingual gyrus	−6	−74	−6	117	−5.12
4	Right inferior temporal gyrus	54	−62	−14	166	−5.25
5	Right insula	40	16	−4	804	−6.95
6	Left insula	−44	6	0	212	−4.95
7	Left posterior cingulate cortex	−6	−52	4	332	−5.30
8	Right superior temporal gyrus	68	−18	12	129	−5.35
9	Right precentral gyrus	44	−22	62	649	−6.44
		50	−8	32	82	−4.81
10	Left paracentral lobule	−10	−30	68	150	−4.83
		−10	−40	54	383	−6.64
11	Left anterior cingulate cortex	−2	28	36	383	−5.64
12	Left median cingulate gyrus	0	−8	50	82	−4.27
13	Right supplementary motor area	4	16	56	148	−5.99

*Abbreviations*: *IGA* internet gaming addiction, *MNI* Montreal Neurological Institute, *MK* kurtosis metrics.

**Table 4:** **Summary of differences in axial kurtosis (K**_**//**_ **) between the IGA and control groups**

	Peak MNI coordinate region	Peak MNI coordinates			Number of cluster voxels	Peak ***t*** value
		x	y	z		
1	Right middle occipital gyrus	50	−68	−16	130	−4.72
2	Right cerebellum anterior lobe	2	−40	−10	329	−5.03
		2	−56	−14	94	−5.64
3	Right precuneus	2	−62	16	336	−5.45
4	Right insula	42	−14	6	92	−4.32
5	Right thalamus	4	−8	6	82	−5.25
6	Left thalamus	−32	−24	2	139	−6.23
7	Right postcentral gyrus	68	−18	14	126	−5.01
8	Right posterior cingulate cortex	0	−40	22	174	−5.62
9	Right supplementary motor area	4	16	54	309	−5.47
10	Left median cingulate gyrus	−10	−40	54	205	−5.44
11	Right precentral gyrus	36	−24	68	342	−5.78
12	Left precentral gyrus	−20	−24	64	117	−5.04

*Abbreviations*: *IGA* internet gaming addiction, *MNI* Montreal Neurological Institute.

**Table 5:** **Summary of differences in radial kurtosis (K**_**⊥**_ **) between the IGA and control groups**

	Peak MNI coordinate region	Peak MNI coordinates			Number of cluster voxels	Peak ***t*** value
		x	y	z		
1	Right inferior temporal gyrus	44	-42	-28	129	-5.57
		56	-60	-14	158	-5.03
2	Right orbitofrontal cortex	20	14	-20	87	-4.30
3	Right fusiform gyrus	-6	-36	-2	225	-5.78
4	Left insula	-44	8	0	102	-5.28
5	Right insula	40	16	2	288	-6.53
		40	-14	6	244	-6.43
6	Left anterior cingulate cortex	-2	36	30	310	-5.62
7	Left median cingulate gyrus	-8	-4	48	399	-5.21
		-8	-40	54	176	-6.18
8	Right postcentral gyrus	38	-30	50	307	-5.29
9	Left paracentral lobule	-12	-30	68	84	-4.78
10	Right posterior cingulate cortex	8	-54	4	166	-4.72

*Abbreviations*: *IGA* internet gaming addiction, *MNI* Montreal Neurological Institute.

### Correlations between differences in GM microstructure and IGA severity

CIAS scores significantly and positively correlated with MK values in the left PCC and with K_⊥_ values in the right PCC (left PCC, MK: r = 0.478, *P* = 0.045; right PCC, K_⊥_: r = 0.497, *P* =0.036).

### VBM analysis

**Figure 4:**
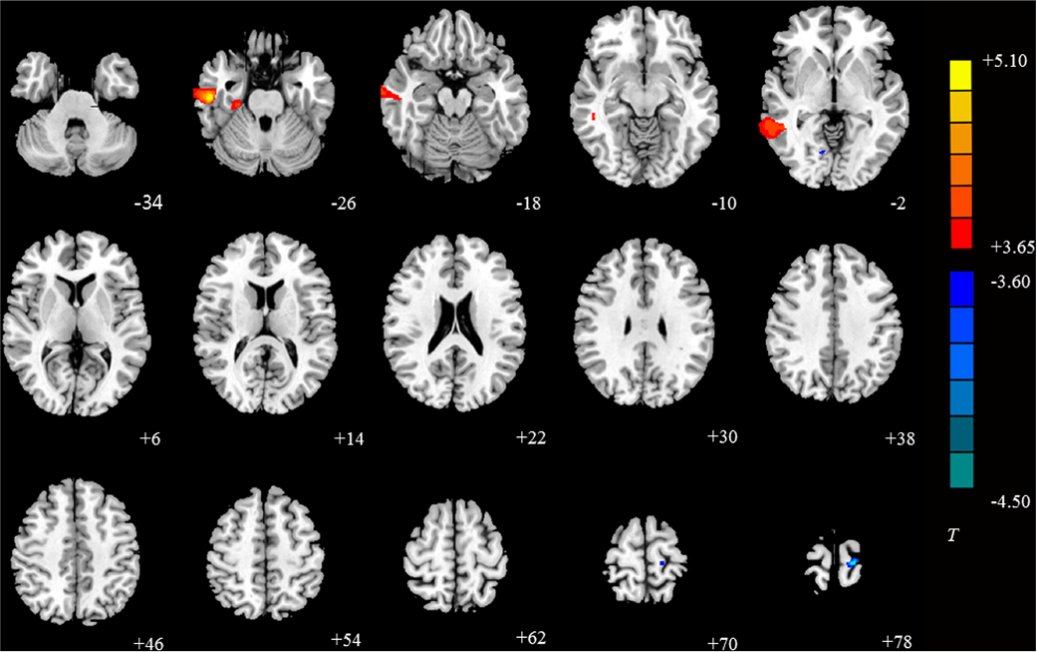
**Significant differences in GM volume between IGA and HC subjects.** The results show IGA subjects had higher GM volume in the right inferior temporal gyrus, right parahippocampal gyrus, and right middle temporal gyrus, and lower GM volume in the left precentral gyrus (*P* <0.001, cluster size was set at >100 voxels). T-values are color-coded on the right. Blue indicates IGA group < HC, yellow indicates IGA group > HC. *The left part of the figure represents the patient’s right side. *HC, healthy control. IGA, internet gaming addiction.

VBM analysis showed that IGA subjects had higher GM volume in the right inferior and middle temporal gyri and the right parahippocampal gyrus, and lower GM volume in the left precentral gyrus compared with controls (Figure 4 and Table 6).

**Table 6:** **Summary of GM volume changes in the IGA group compared with the control group**

	Peak MNI coordinate region	Peak MNI coordinates			Number of cluster voxels	Peak T value
		x	y	z		
1	Right inferior temporal gyrus	54	−19	−27	1012	5.39
2	Right parahippocampal	27	−24	−24	198	4.07
3	Right middle temporal gyrus	48	−45	−3	837	4.35
4	Left precentral gyrus	−18	−28	76	190	−4.71

*Abbreviations*: *IGA* internet gaming addiction, *MNI* Montreal Neurological Institute, *GM* gray matter.

## Discussion

As a clinically feasible extension of DTI, in addition to the orientation-dependent apparent diffusion coefficient (ADC), DKI provides additional information regarding tissue substructure [20]. DKI is more sensitive to GM, and thus provides improved GM-WM contrast [31]. Owing to the isotropic water diffusion that occurs in GM, diffusivity parameters derived from DTI have limited clinical value. DKI-derived parameters can overcome this limitation. MK is a dimensionless parameter that reflects the degree of diffusion restriction, while K_//_ and K_⊥_ measure the kurtoses along the directions parallel and perpendicular to the principal diffusion direction, respectively. In contrast to conventional DTI parameters, MK is regarded as an index of tissue-microstructure complexity, such as density, orientation, degree of cell-membrane organization, axon sheaths, and myelin layers. Accordingly, parameters derived from DKI are highly sensitive to changes in microstructural tissue organization that occur during postnatal maturation of the normal brain [32, 33]. Our results showed that the primary DKI parameters (MK, K_//_, and K_⊥_) showed similar tendencies, but still had obvious differences depending on brain region. In GM, apart from the addition of basal dendrites and modification in tissue water content and cell packing density, it is known that changes in cortical cytoarchitecture affect how water diffuses [34, 35]. MK is known to be highly sensitive in detecting general differences, but directional kurtoses are capable of providing more specific information regarding diffusion restriction along a particular direction. For instance, K_⊥_ is highly sensitive to myelination [22]. More biological evidence is needed for a complete understanding of these specific observations. DTI studies have primarily reported altered WM integrity in subjects with IGA [14, 36], whereas only a few studies have reported GM abnormalities in IGA subjects using VBM. IA adolescents have been demonstrated to possess lower GM volume in the left ACC, left PCC, left insula, and left lingual gyrus [15]. Furthermore, Hong et al. confirmed that adolescent boys with IA had significantly smaller cortical thicknesses in the right lateral OFC than controls, supporting the hypothesis that changes in the OFC of adolescents with IA was a neurobiological marker of addiction-related disorders in general [37]. Yuan et al. also demonstrated lower GM volume in bilateral dorsolateral prefrontal cortex (DLPFC), SMA, OFC, cerebellum, and the left rostral ACC (rACC) in adolescents with IA. GM volumes in the DLPFC, rACC, and SMA were significantly correlated with IA duration. Another study suggested that long-term IA could result in alterations in brain structure, which is likely to contribute to the chronic dysfunction observed in IA [38]. More specifically, compared with healthy subjects, IGA individuals were observed to have significant GM atrophy in the right OFC, bilateral insula and right SMA; GM volumes in the right OFC and bilateral insula were significantly positively correlated with the severity of IGA [39].

In the present study, preliminary results show a decrease in DKI parameters in the GM of IGA subjects, indicating the spread of GM damage across the brain. GM abnormalities that were found in the OFC, insula, cingulate gyrus, SMA, ACC, PCC, precentral gyrus, cerebellum, and precuneus were consistent with previous findings [1, 15, 38–40]. Most of these areas were mentioned in a model proposed by Volkow et al. [41], who presented a model in which addiction emerges as an imbalance in information processing and integration among various brain circuits and functions. The ACC has been shown to be essential for motor control, cognition, and motivation [42], to be associated with self-control [43], and to encode the reward value during decision-making [44]. The OFC has been shown to be involved in processing emotions and cravings, in maladaptive decision-making processes, and in engaging in compulsive behaviors, each of which is integral to addiction [45]. SMA-mediated cognitive control [46] and the cingulate gyrus both contribute significantly to integrating sensory information and monitoring conflict [47]. The lingual gyrus has been linked to regulation of emotional behavior, which is a major concern in IA [15]. The precuneus possesses a critical function in processing visual spatial information and spatially guided behavior [48], and the precentral gyrus (primary motor cortex) has recently been implicated in the mechanisms underlying enhanced motivational drive for a drug [49]. Subtle modifications in the structure of various GM regions in IGA subjects may contribute to the behaviors resulting from excessive online gaming, such as issues associated with impulse control, behavioral inhibition, executive functioning, attention, and general cognitive functioning. However, as a cross-sectional study, the results of this study do not demonstrate whether the different GM microstructures found in IGA subjects preceded the development of IGA or were the consequence of gaming addiction. In addition, the changes in the parameters derived from DKI may be caused by other pathologies (such as depression or anxiety). As we listed in Tables 1 and 2, the IGA group possessed higher SAS, SDS, and BIS-11 scores than the controls, which may suggest psychiatric disorders such as depression or anxiety that could also lead to gray matter abnormalities [50, 51]. It remains unknown whether IGA is truly a unique phenomenon or simply the symptoms of underlying mental health problems [52]. Therefore, the underlying pathophysiology of the interesting findings in DKI parameters observed in GM requires further study.

Lower DKI parameters were also observed in the inferior and superior temporal gyri, middle occipital gyrus, and fusiform gyrus. The fusiform gyrus is a section of the temporal and occipital lobes that functions in processing of color information, face and body recognition, word recognition, and within-category identification, as well as the perception of emotions in facial stimuli [53–55]. The temporal regions are involved in auditory processing, comprehension, and verbal memory, whereas the occipital regions control visual processing. Recently, Han et al. [40] reported that GM volumes in the inferior temporal cortex and occipital lobe were diminished in both online game addiction and pro-gamers when compared with healthy volunteers. These two regions could be damaged by harmful visual stimuli, probably owing to the excessive exposure to visual and auditory stimulation during online games.

In the present study, the IGA group exhibited lower MK values in the left PCC, lower K_//_ values in the right PCC, and lower K_⊥_ values in the right PCC. Further, positive correlations between CIAS scores and MK value were observed in left PCC and between CIAS scores and K_⊥_ value in right PCC. The PCC participates in visual-spatial orientation and processing self-related information, is part of the default mode network [56], and often receives input via multiple sensory channels (e.g., visual, tactile, auditory, and proprioceptive). This may explain its powerful involvement in neural cue reactivity in the presence of visual and other modality stimuli. Using the reliable multisensory cue-induced activation of PCC as a biomarker may contribute to the development of successful high-tailored, subject-specific therapeutic strategies [57]. Although most previous studies have reported functional and structural abnormalities in the left PCC [15, 36, 58], we also found abnormalities in the right PCC. This inconsistency may be owing to the sensitivities of the different DKI parameters, but it still calls for further investigation of the exact underlying mechanisms.

Our VBM result was inconsistent with our result from the DKI or those from previous studies. This discrepancy may have resulted from the small sample size, the severity of our subjects’ addiction, or the data-analysis methods. Additionally, the differences in the DKI parameters between IGA subjects and controls could act as a potential precursor of further modifications in the gray matter not yet expressed in the VBM-based analysis. Further studies are required to clarify this pointe.

There are several potential limitations of the present study. First, the relatively small sample size may limit the generalization of the results; more biological evidence is warranted to understand these specific observations. Second, because the study focused on only one subtype of IA, the results may not extend to the other IA subtypes. Third, a GM mask was applied for the analysis using segmented tissue maps from high-resolution 3D-SPGR images. The results of the mask depended on the segmentation results, and further methodological advances might improve the current correction technique. Last, potential confounding factors such as levels of physical activity and school performance were not excluded. Future prospective studies should therefore focus on clarifying the causal relationship between IGA and psychological measures.

## Conclusion

DKI can detect subtle differences in GM microstructure between IGA and HC individuals. The decrease of K_⊥_, K_//_, and MK values in IGA subjects indicated multiple changes in brain microstructure, which may be implicated in the underlying pathophysiology of IGA. Furthermore, the DKI model can provide sensitive imaging biomarkers for assessing the severity of IGA.
